# Supplementary figures and images for: The PHD finger protein Spp1 has distinct functions in the Set1 and the meiotic DSB formation complexes
Source: PLoS Genet. 2018 Feb 14;14(2):e1007223. doi: 10.1371/journal.pgen.1007223 (PMC5828529; doi:10.1371/journal.pgen.1007223)

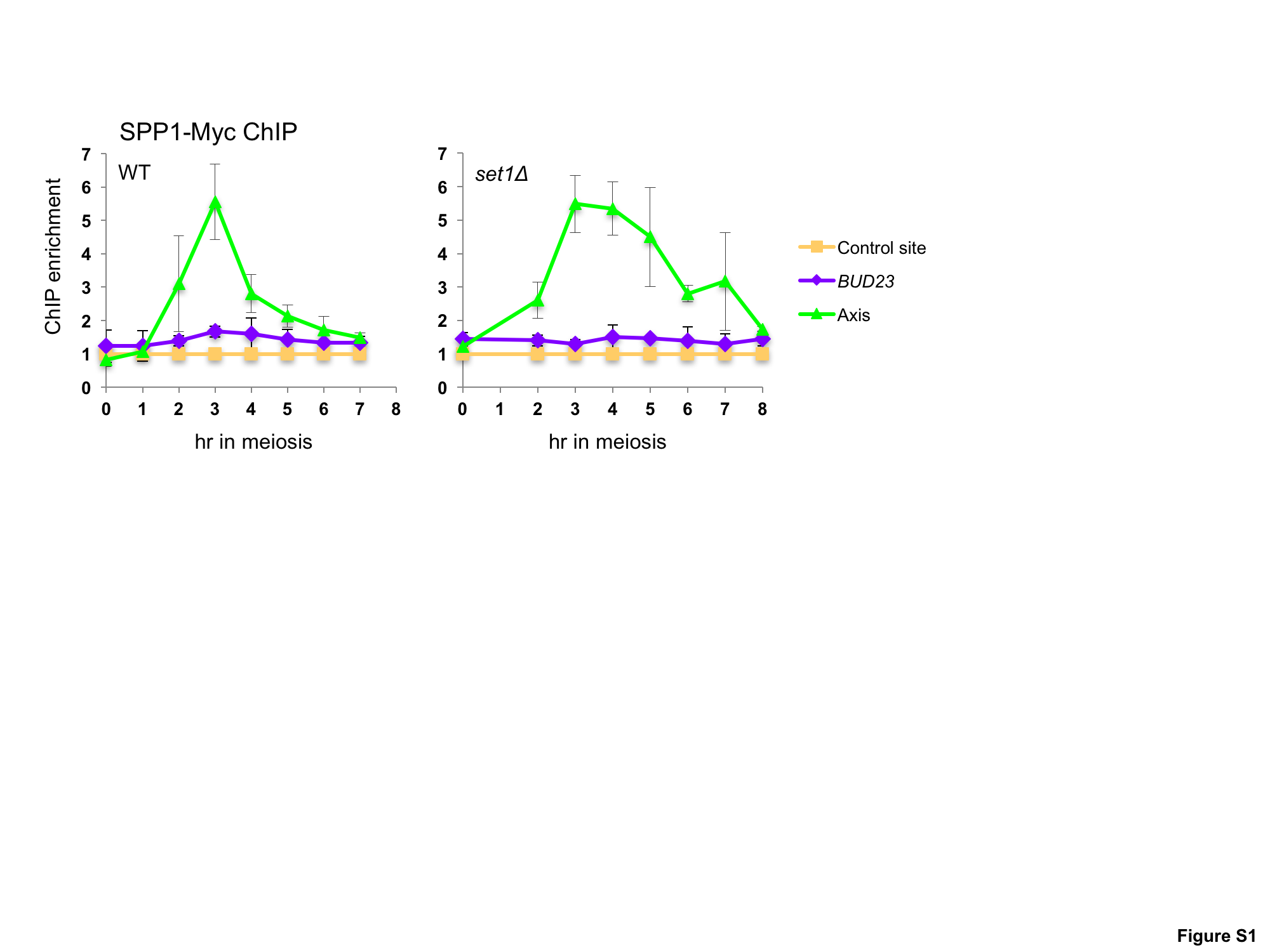

Supplement: S1 Fig — ChIP-qPCR of Spp1 during meiosis in SET1 (VBD1187) or set1∆ (VBD1209) diploids. Values represent mean ± range of two independent experiments. (TIFF) [file pgen.1007223.s001.tiff]

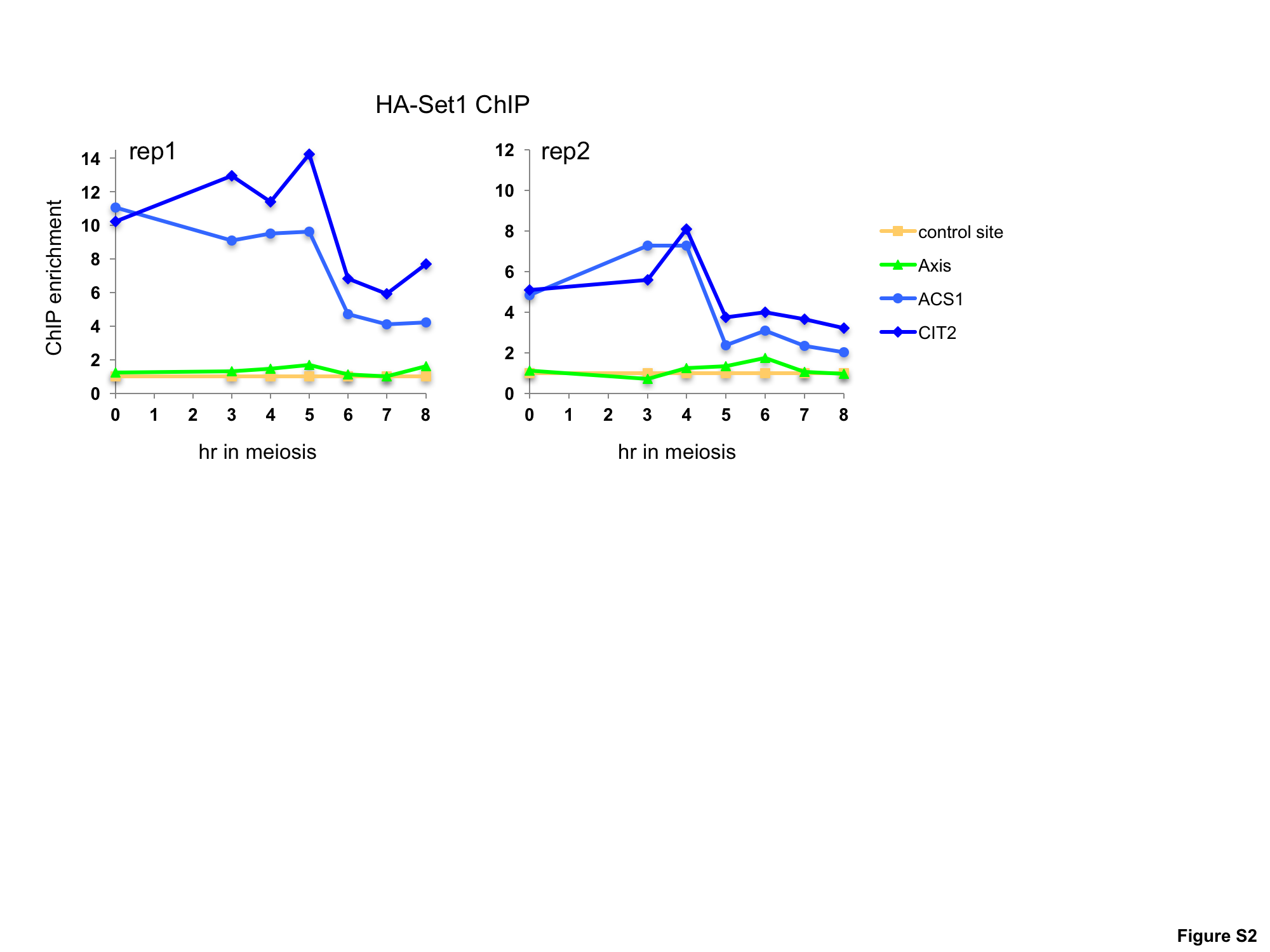

Supplement: S2 Fig — ChIP-qPCR of HA-Set1 during meiosis (VBD1378 strain). Two biological replicates are shown. (TIFF) [file pgen.1007223.s002.tiff]

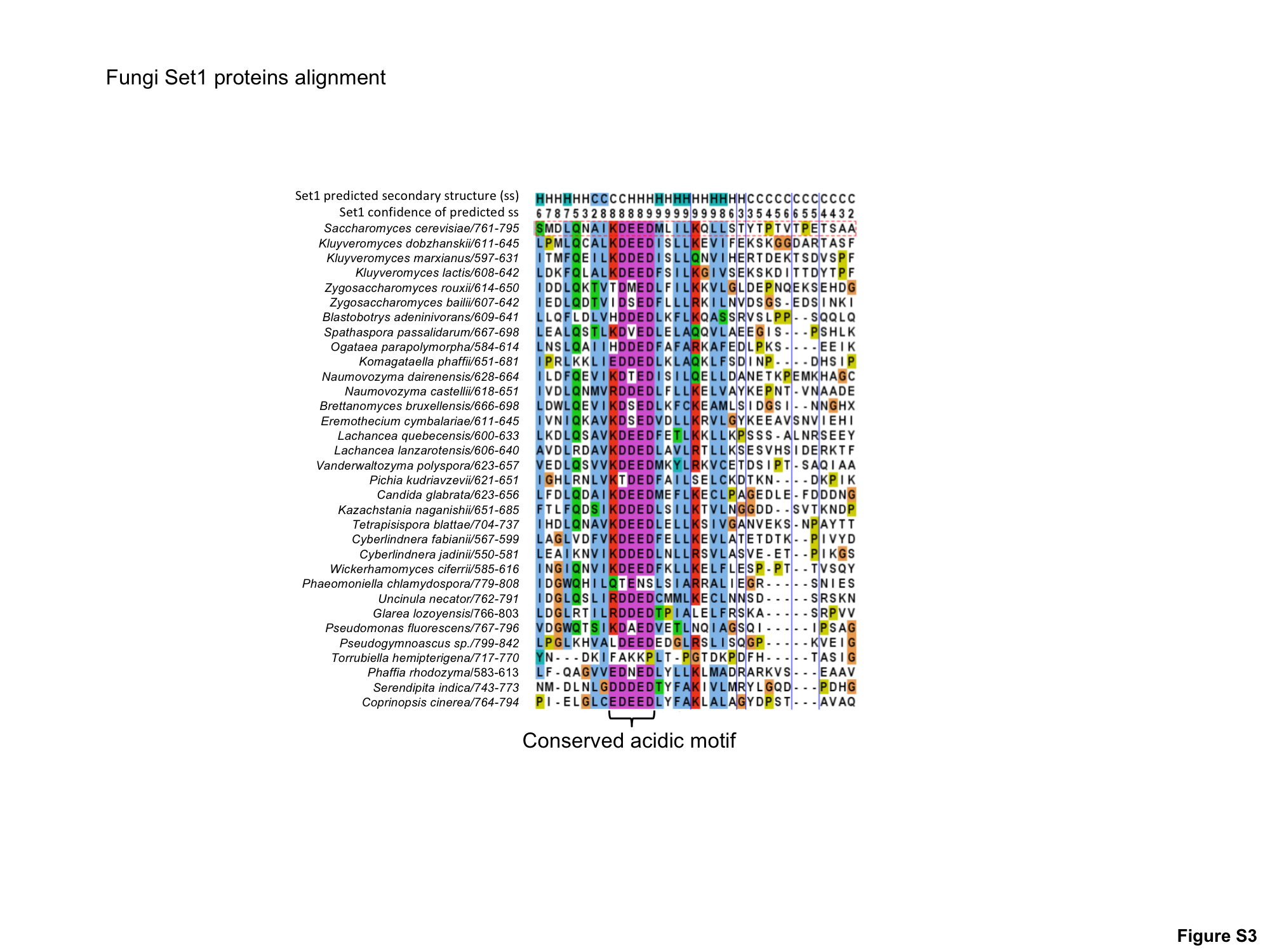

Supplement: S3 Fig — Above is indicated the predicted structure (H: or C:) with its confidence level. (TIFF) [file pgen.1007223.s003.tiff]

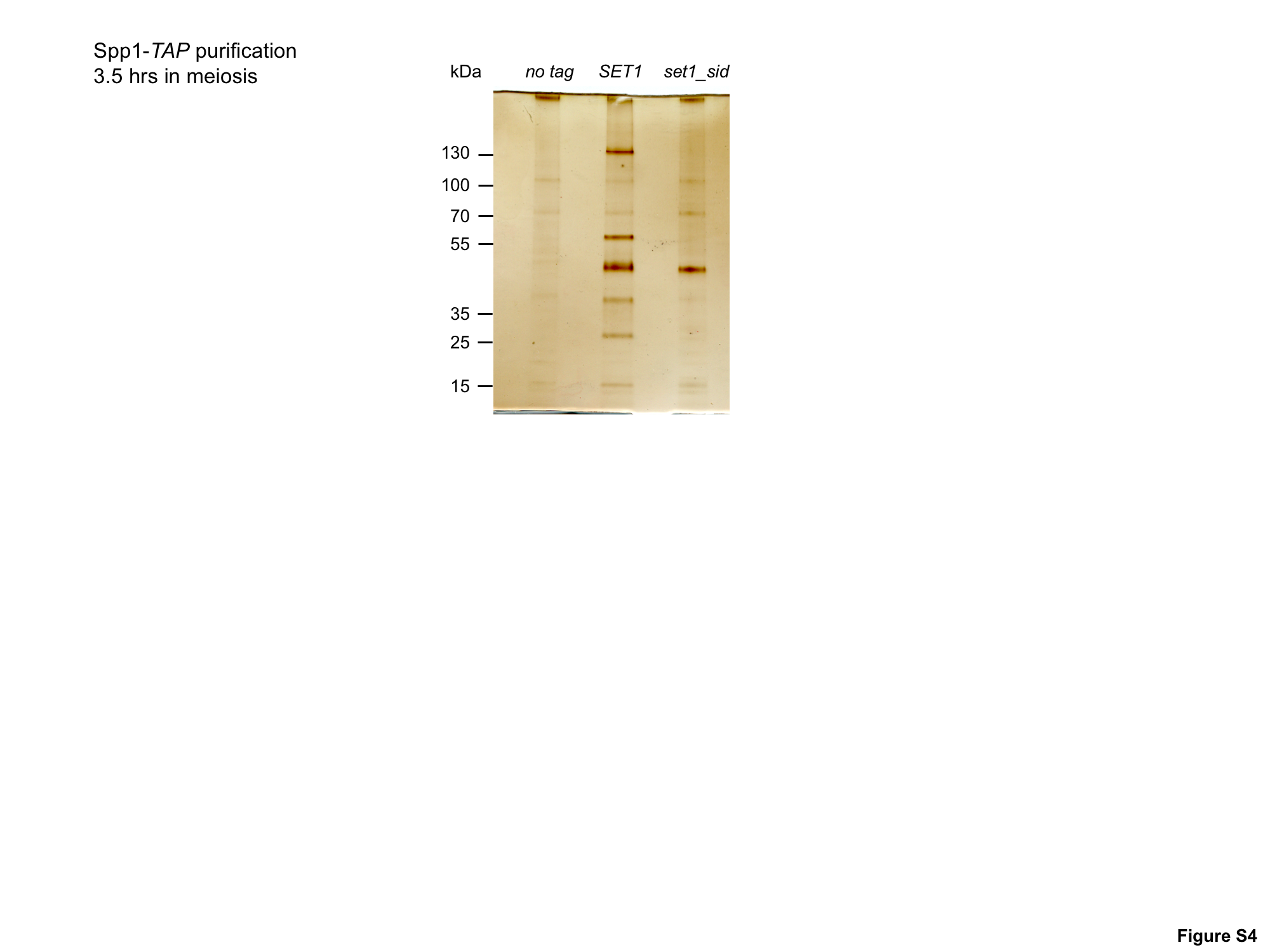

Supplement: S4 Fig — Silver-stained gel of TAP eluates performed at 3.5 hrs in meiosis in an untagged (ORD7339), SPP1-TAP SET1 (VBD1266) or SPP1-TAP set1_sid (VBD1877) strain. Untagged and Spp1-TAP samples are from the same gel as in Fig 1B. (TIFF) [file pgen.1007223.s004.tiff]

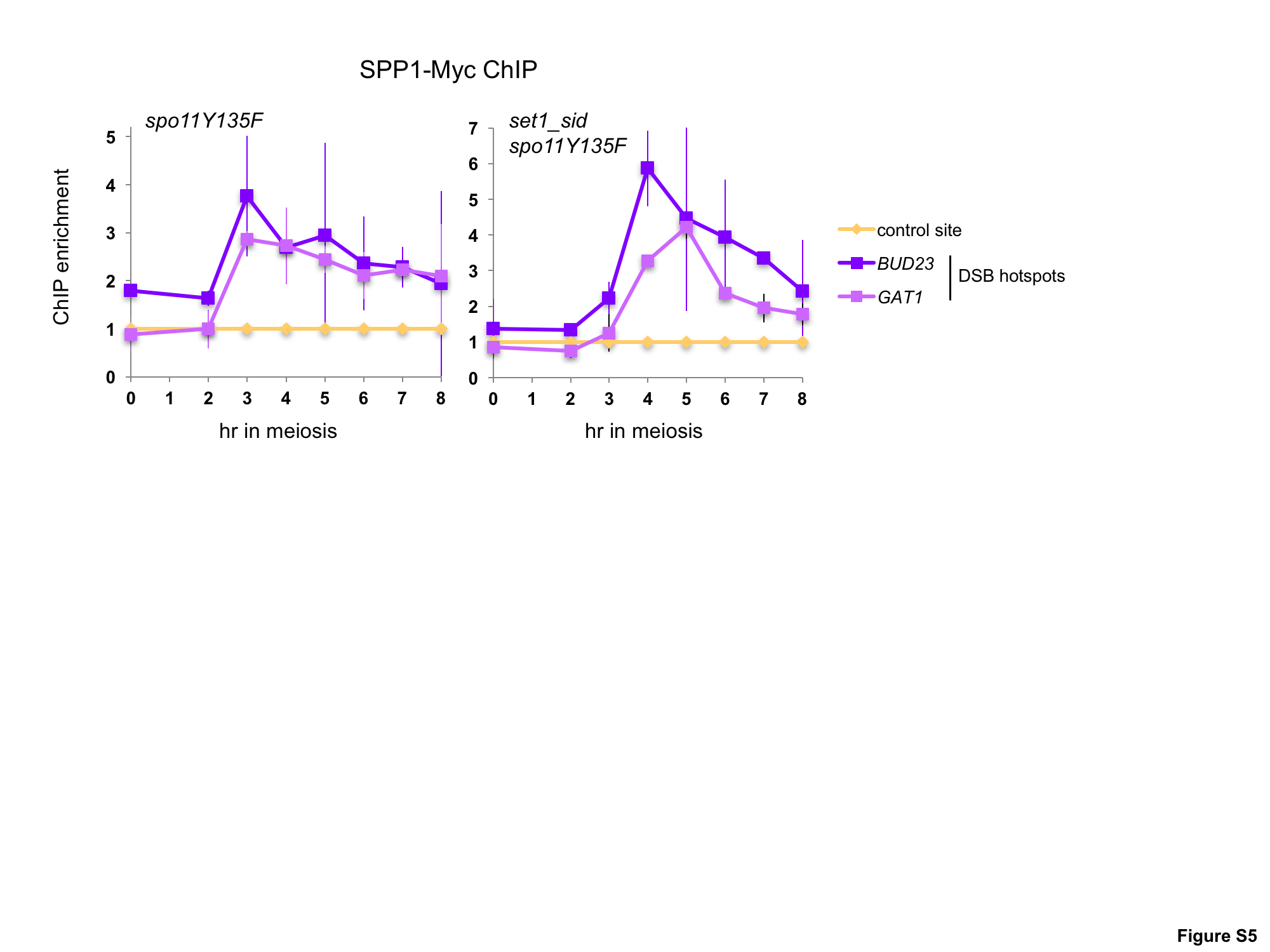

Supplement: S5 Fig — ChIP-qPCR of Spp1 during meiosis in spo11Y135F (VBD1248) or spo11Y135F set1_sid (VBD1944) diploids. Values represent mean ± range of two independent experiments. (TIFF) [file pgen.1007223.s005.tiff]

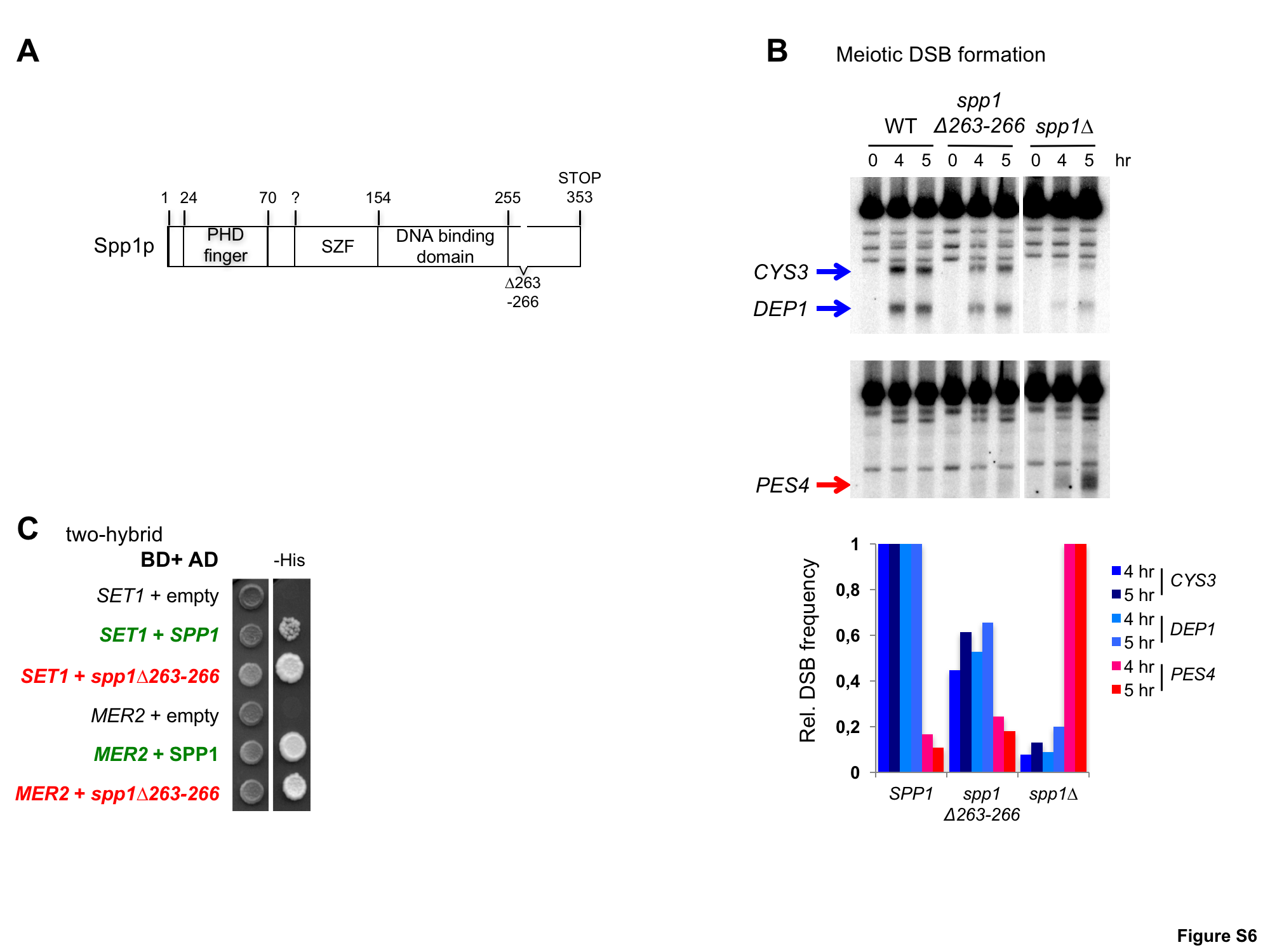

Supplement: S6 Fig — (A) Schematic structure of the Spp1 protein sequence with the position of ∆263–266 mutation. (B) Meiotic DSB formation in dmc1∆ cells by Southern blot at CYS3 and DEP1 DSB (upper panel), or at the spp1∆-specific PES4 DSB (lower panel). WT: VBD1689; spp1∆263–266: VBD1737; spp1∆: VBD1748. Graph shows the DSB quantification relative to the level in WT (for CYS3, DEP1) or spp1∆ cells (for PES4 site). DSB were quantified at the 5 hr time point. (C) Two-hybrid interaction between Spp1∆263–266 and Set1 or Mer2 proteins. Growth on–His indicates an interaction between the two tested proteins. (TIFF) [file pgen.1007223.s006.tiff]

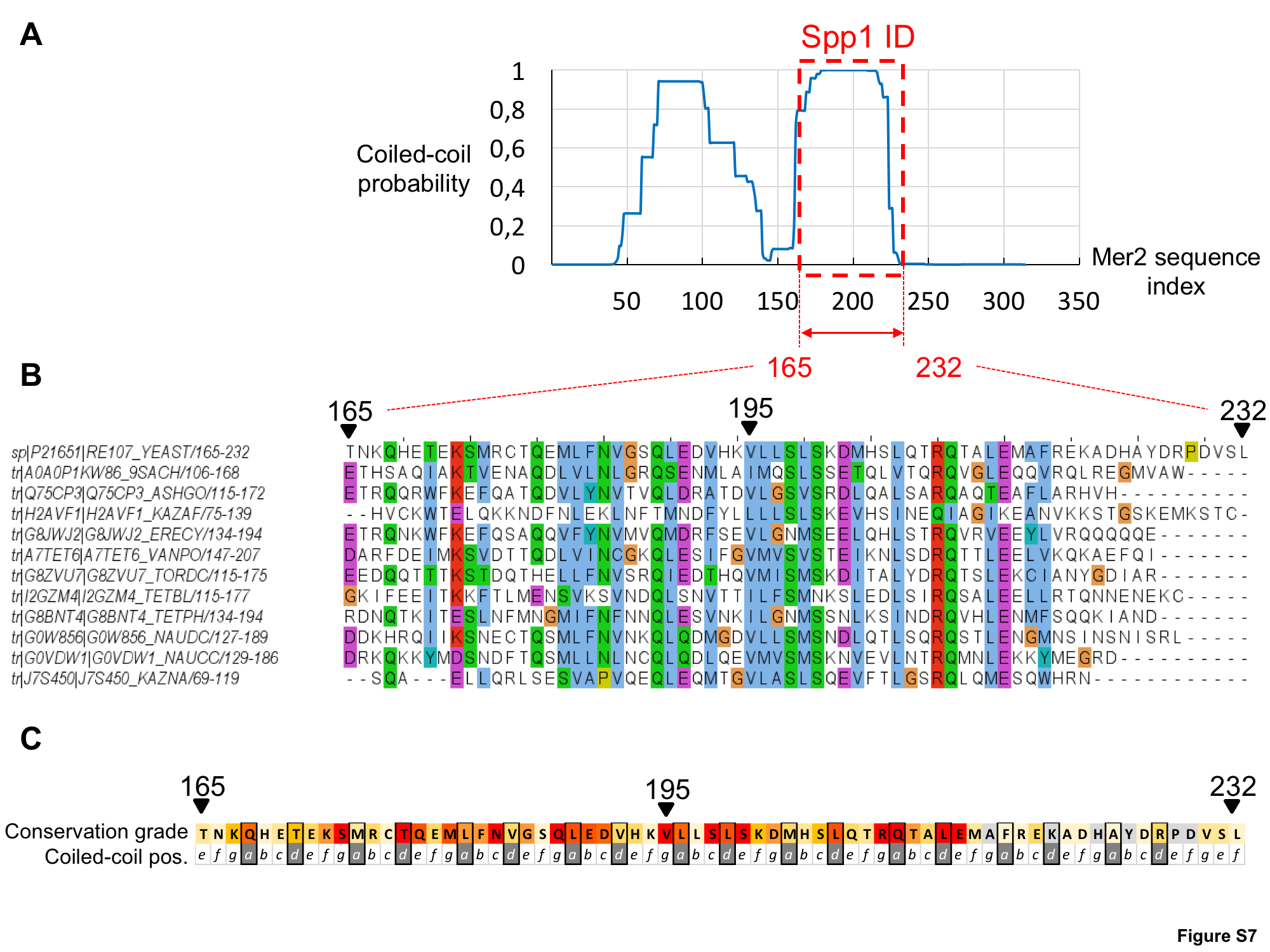

Supplement: S7 Fig — (A) Coil-coiled prediction probability vs Mer2 sequence using HHblits to build the multiple sequence alignment of Mer2 and Pcoil to run coiled-coil predictions [61]. The dashed red square indicates the delimitations of the minimal Spp1 interacting domain (Spp1 ID) of Mer2 as reported [17]. (B) Multiple sequence alignment of Mer2 homologs focused on the region comprising the Spp1 interacting domain (segment 165–232 of Mer2). (C) Sequence of the Spp1 interacting domain of Mer2, with the top sequence color code of reporting the conservation grade from gray (variable) to red (highly conserved) as calculated by Consurf server [62] and the bottom sequence reporting the predicted location of residues in the coiled-coil heptad, with positions “a” and “d” matching the most buried residues indicated by squared shaded boxes. (TIFF) [file pgen.1007223.s007.tiff]

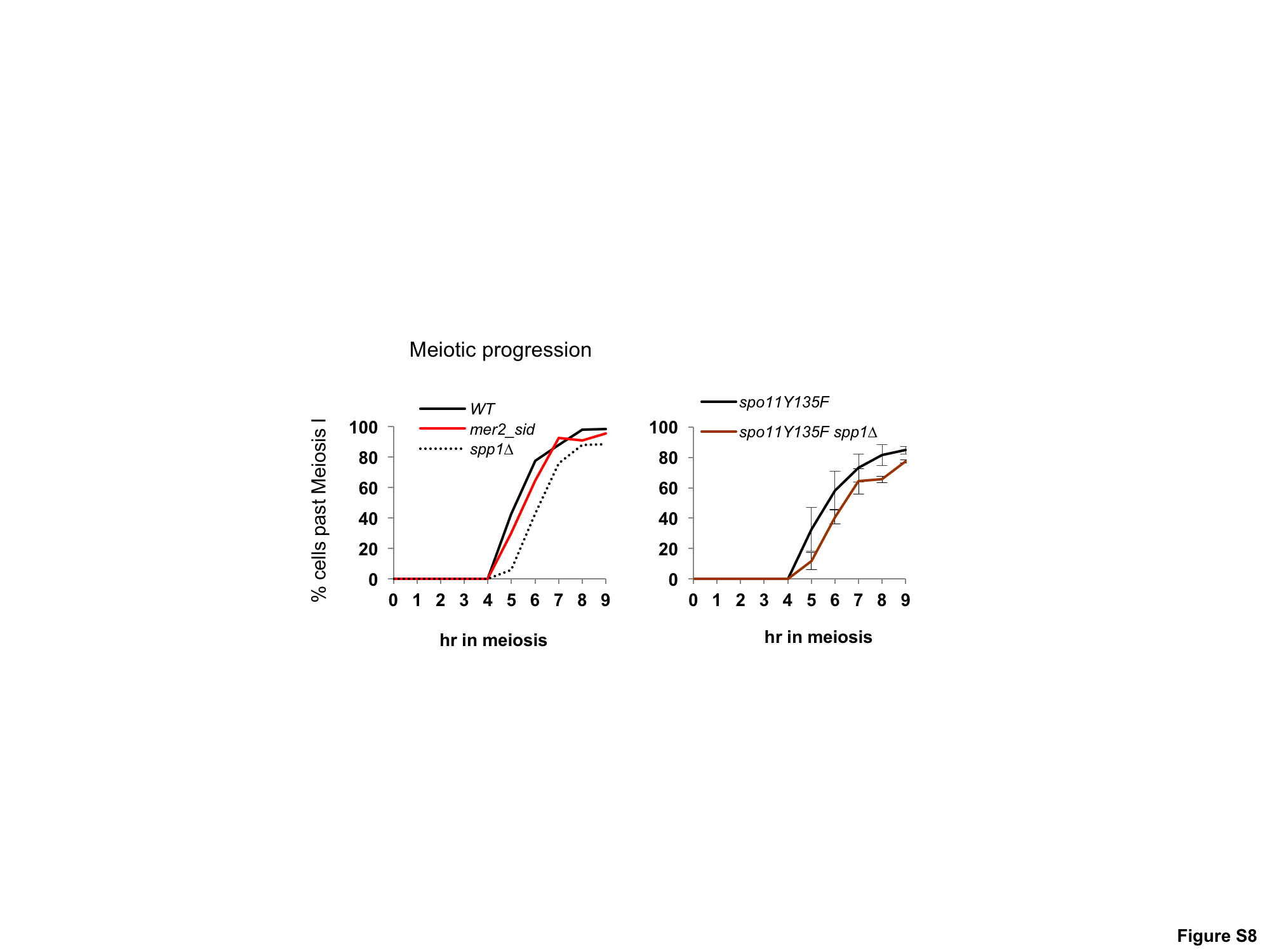

Supplement: S8 Fig — Meiotic progression as assessed by DAPI staining of strains with the indicated genotype. WT: ORD7339; mer2_sid: VBD1880; spp1∆: VBD1769; spo11Y135F: VBD1291; spo11Y135F spp1∆: VBD1233. Error bars indicate range of two independent experiments. (TIFF) [file pgen.1007223.s008.tiff]
