# Supplementary material for: The PHD finger protein Spp1 has distinct functions in the Set1 and the meiotic DSB formation complexes
Source: PLoS Genet. 2018 Feb 14;14(2):e1007223. doi: 10.1371/journal.pgen.1007223 (PMC5828529; doi:10.1371/journal.pgen.1007223)
Supplement: S2 Table — (DOCX) [file pgen.1007223.s010.docx]

**S2 Table: Genotypes of strains used in this study.**

All strains are of the SK1 background and are *ura3/” lys2/” ho::LYS2/”* unless otherwise stated.

Diploids: The MATa parent is indicated first.

Strains are listed in the order they first appear in the text.

**Strain name Genotype**

ORD7339 *a/l leu2-R/leu2-K arg4-nsp,bgl/’’* (Sommermeyer et al., 2013)

VBD1266 *a/l leu2/” arg4-nsp,bgl/’’ SPP1-TAP::URA3/”*

VBD1220 *a/l leu2/’’ arg4/’’ SPP1-Myc13::hphMX/” mer2∆::URA3/”* (Sommermeyer et al., 2013)

VBD1209 *a/l leu2/’’ arg4/’’ SPP1-Myc13::hphMX/” set1∆::kanMX/”* (Sommermeyer et al., 2013)

VBD1187 *a/l leu2/’’ arg4/’’ SPP1-Myc13::hphMX/”* (Sommermeyer et al., 2013)

VBD1378 *a/l leu2/’’ arg4/’’ HA6-SET1/”*

VBD1399 *a/l leu2/’’ arg4/’’ SWD1-HA3::kanMX/”*

VBD1395 *a/l leu2/’’ arg4/’’ SPP1-Myc13::hphMX/” MER2-His6-Flag3::kanMX/”*

VBD1400 *a/l leu2/’’ arg4/’’ SPP1-Myc13::hphMX/” SWD1-HA3::kanMX/”*

VBD1401 *a/l leu2/’’ arg4/’’ SPP1-Myc13::hphMX/” MER2-His6-Flag3::kanMX/” SWD1-HA3::kanMX/”*

VBD1745 *a/l leu2/’’ arg4/’’ SPP1-TAP::URA3/” MER2-His6-Flag3::kanMX/” SWD1-HA3::NatMX/”*

VBD1742 *a/l leu2/’’ arg4/’’ MER2-His6-Flag3::kanMX/” SWD1-HA3::NatMX/”*

VBD1402 *a/l leu2/” arg4-nsp,bgl/’’ MER2-TAP::URA3/”*

VBD1836 *a/l leu2/’’ arg4/’’ SPP1-TAP::URA3/” MER2-His6-Flag3::kanMX/” SWD1-HA3::NatMX/” set1_sid/”*

VBD1868 *a/l leu2/’’ arg4/’ SPP1-Myc13::hphMX/” set1_sid/”*

ORD7354 *a/l leu2/’’ arg4/’ his4X” dmc1∆::LEU2* (Sommermeyer et al, 2013)

VBD1854 *a/l leu2/’’ arg4/’ set1_sid/” dmc1∆::LEU2*

VBD1748 *a/l leu2/’’ arg4/’ spp1∆::kanMX/” dmc1∆::LEU2*

ORD9624 *a/l leu2/’’ arg4/’ set1∆::kanMX/” dmc1∆::LEU2* (Sommermeyer et al, 2013)

VBD1248 *a/l leu2/’’ arg4/’’ SPP1-Myc13::hphMX/” spo11(Y135F)- His6-Flag3::NatMX/”* (Sommermeyer et al, 2013)

VBD1944 *a/l leu2/’’ arg4/’’ set1_sid/” SPP1-Myc13::hphMX/” spo11(Y135F)- His6-Flag3::NatMX/”*

ORT4601 *MATl leu2-K arg4-nsp,bgl*

ORT4784 *MATl leu2-K arg4-nsp,bgl set1∆::kanMX*

VBH152 *MATl leu2-K arg4-nsp,bgl spp1∆::kanMX*

VBH1881 *MATl leu2-K arg4-nsp,bgl set1_sid*

VBH1972 *MATl leu2-K arg4-nsp,bgl set1_sid spp1∆::kanMX*

VBH1419 *MATl ura3 ho::hisG leu2::hisG HIS4::LEU2-(BamH1; +ori) spp1W45A-3'UTR-hphMX*

VBH2021 *MATl leu2-K arg4 set1_sid spp1W45A-3'UTR-hphMX*

VBD1852 *a/l leu2/’’ arg4/’ mer2_sid-His6FLAG3-kanMX/” Swd1-3HA-NatMX6/” SPP1-TAP::URA3/”*

VBD1251  *a/l leu2/’’ arg4/’’ MER2-His6-Flag3::kanMX/”*  (Sommermeyer et al, 2013)

VBD1843  *a/l leu2/’’ arg4/’’ mer2_sid-His6-Flag3::kanMX/”*

VBD1924 *a/l leu2/’’ arg4/’’ mer2_sid/” SPP1-Myc13::hphMX/”*

VBD1879 *a/l leu2/’’ arg4/’ mer2_sid/” dmc1∆::LEU2*

VBD1880 *a/l leu2/’’ arg4/’ mer2_sid/”*

VBD1769 *a/l leu2/’’ arg4/” spp1∆::kanMX/”*

VBD1291 *a/l leu2/’’ arg4/” spo11Y135F-His6Flag3::NatMX*

VBD1233 *a/l leu2/’’ arg4/” spp1∆::kanMX/” spo11Y135F-His6Flag3::NatMX*

VBD1877 *a/l leu2/leu2 arg4-nsp,bgl/’’ set1_sid/” SPP1-TAP::URA3/”*

VBD1689  *a/l leu2/’’ arg4/” SPP1-3'UTR-hphMX” dmc1∆::LEU2*

VBD1737  *a/l leu2/’’ arg4/” spp1∆263-266-3'UTR-hphMX” dmc1∆::LEU2*

Sommermeyer, V., Beneut, C., Chaplais, E., Serrentino, M.E., and Borde, V. (2013). Spp1, a Member of the Set1 Complex, Promotes Meiotic DSB Formation in Promoters by Tethering Histone H3K4 Methylation Sites to Chromosome Axes. Molecular Cell *49*, 43-54.
